# Protocol for analyzing slow cortical dynamics in mouse neuronal recordings

**DOI:** 10.1016/j.xpro.2026.104804

**Published:** 2026-08-27

**Authors:** Yuriy Shymkiv, Rafael Yuste

**Affiliations:** 1 Neurotechnology Center, Department of Biological Sciences, Columbia University, New York, NY, USA

**Keywords:** Neuroscience, Cognitive Neuroscience, Computer sciences

## Abstract

Temporal information processing is critical for brain function, supporting neural computations such as novelty detection, adaptation, and temporal normalization. Its disruption is implicated in schizophrenia. We present a protocol for analyzing ongoing neuronal network activity using binwise decoding, trial-to-trial variability analysis, and estimation of network-intrinsic timescales (INTs). We apply these techniques to identify slow dynamics that encode the memory of recent stimuli in neuronal populations in the mouse auditory cortex and in artificial neural networks trained on a novelty-detection task.

For complete details on the use and execution of this protocol, please refer to Shymkiv et al.^1^

## Highlights

• Measuring slow multineuronal activity in cortical networks using binwise decoders
• Characterizing impact of slow network dynamics on trial-to-trial variability
• Intrinsic timescale (INT) metric for analyzing network and neuronal timescales

## Abstract (editor highlights)

Publisher’s note: Undertaking any experimental protocol requires adherence to local institutional guidelines for laboratory safety and ethics.

## Graphical abstract

**Figure:**
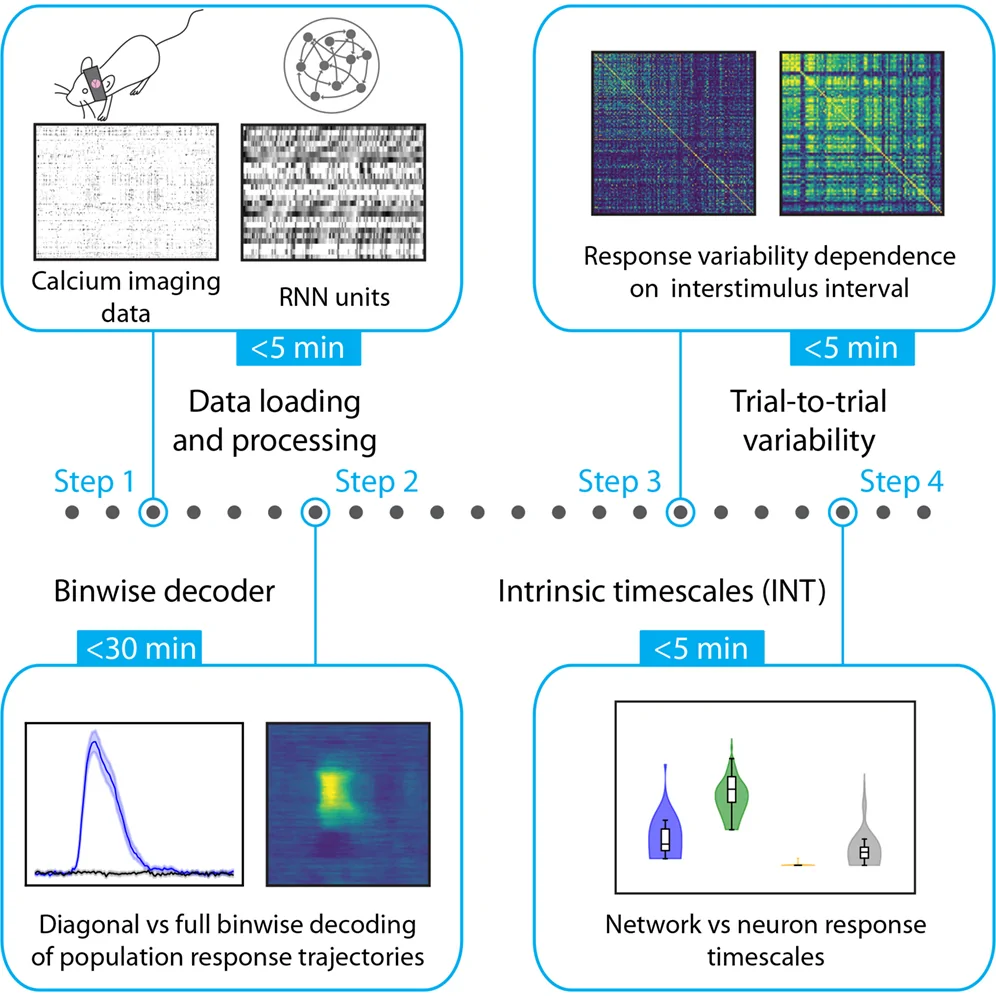

## Before you begin

Retaining information over short timescales is critical for context-dependent computations and normal brain function.^2^ These transient memory processes support low-level computations as well as higher-order cognition and behavior. In sensory cortex, such processes are thought to emerge from recurrent circuit dynamics and contribute to novelty detection, adaptation, and temporal normalization, allowing neuronal responses to be shaped by recent stimuli and ongoing network activity.^3^^,^^4^^,^^5^ Importantly, deficits in temporal processing are observed in patients with schizophrenia and other psychotic disorders, in whom impairments in early sensory processing and predictive integration may contribute to higher-order cognitive dysfunction.^6^^,^^7^^,^^8^

Short-term memory of sensory inputs can be encoded in the activity of individual neurons or neuronal networks through prolonged responses, referred to here as “slow dynamics.”^9^^,^^10^^,^^11^ As a form of system-level hysteresis, these dynamics may reflect sensory inputs or more abstract, computation-related features.^2^^,^^12^^,^^13^ Extended responses in individual neurons are prevalent in higher-order regions such as the prefrontal cortex (PFC) but are less common in sensory cortex.^14^^,^^15^ Nevertheless, temporal sequences of single-neuron activations in sensory cortex can maintain stimulus-related information over time in the form of trajectories through network state space. Combinations of such activity sequences in primary cortex may underlie sustained neuronal activity in higher-order regions.

In this work, we present three methods for analyzing slow dynamics in neurons and networks. First, we describe a binwise stimulus decoder^16^^,^^17^ that reveals how stimulus-related activity evolves over time across the neuronal population. Second, we use trial-to-trial variability^18^^,^^19^ as an indirect measure of context-dependent network states. Third, we apply an autocorrelation-based intrinsic timescale (INT) metric^20^^,^^21^^,^^22^ to quantify the duration of spontaneous and stimulus-evoked activity at both the single-neuron and network levels. We demonstrate these methods using two-photon calcium imaging datasets recorded from the auditory cortex of awake mice during passive presentation of auditory stimuli in an oddball paradigm, as well as synthetic data generated by recurrent neural networks (RNNs) trained to perform novelty detection or stimulus recognition as a control task.

The three analyses measure a common quantity—the timescale over which ongoing circuit activity retains memory of recent stimuli—but approach it from complementary perspectives. The intrinsic timescale (INT) quantifies this memory as the duration over which activity remains displaced from baseline; we compute it for individual neurons and, by extending the metric to the population level, for the network. The binwise decoder characterizes memory through the persistence and organization of stimulus information: the diagonal decoder measures how long stimulus identity remains decodable, whereas the full (cross-temporal) decoder tests whether the population code is static (sustained) or dynamic (following an evolving trajectory). The trial-to-trial variability analysis quantifies memory functionally: when stimuli are presented in random sequences, a persistent network state induced by preceding stimuli affects the current response, increasing trial-to-trial variability; this effect diminishes as the inter-stimulus interval increases. Taken together, the three analyses converge on a memory timescale and cross-validate one another. Comparing the single-neuron and network measures can help distinguish whether slow network dynamics arise from sustained firing in individual neurons or from sequences of transient single-neuron activations.

### Innovation

This protocol combines three complementary computational analyses of slow network dynamics at single-cell resolution and applies them identically to datasets from two-photon calcium imaging and RNNs.

First, we track how stimulus information evolves across the population using a binwise cross-temporal decoder, an established approach in coarse-scale neuroimaging and electrophysiology.^16^^,^^17^ We use this decoder to identify sustained population states within an evolving trajectory.

Second, we use trial-to-trial response variability to measure the persistence of the network’s stimulus-evoked state, building on the established relationship between such variability and the network’s ongoing state.^18^^,^^19^ The innovation lies in estimating the timescale from the recovery of trial-to-trial correlations as the inter-stimulus interval increases.

Third, we apply intrinsic timescale (INT) analysis, which has previously been used to estimate timescales from individual-neuron activity^20^ or pooled population activity.^22^ We introduce a network INT estimated from the autocorrelation of the population’s displacement from its baseline state and compare it with single-neuron INTs. This comparison allows us to test the hypothesis that a sequence of transiently active neurons with individually short timescales can keep the population displaced from baseline, producing a long network INT that cannot be detected using single-neuron or pooled-rate measures. RNNs trained on different tasks provide lower and upper reference ranges for this comparison: networks trained on a memory-independent task define the lower range, networks trained on a memory-dependent task define the upper range, and the cortical network timescale falls between them.

### Hardware

Analysis can be performed using the Anaconda Prompt on Windows or a terminal on Linux or macOS. A system with at least 16 GB of RAM and four CPU cores is sufficient for the calcium imaging analyses and the RNN control-input datasets. The RNN oddball-input datasets are larger and are loaded entirely into memory, so analyzing them requires substantially more RAM (at least 64 GB is recommended). This protocol was tested on a Windows 10 machine with an Intel Core i9-14900K processor and 128 GB of RAM.

### Software preparation

**Timing: <15 min**

This section describes how to set up the software environment by installing Miniconda, creating a dedicated Python environment, and installing the packages required to run the analysis pipeline.

1. Install Miniconda from https://www.anaconda.com/.
2. Open the Anaconda Prompt on Windows or a terminal on Linux or macOS, create a new environment, and install the required Python packages listed in the key resources table.

$ conda create -n slowdyn python=3.12.12

$ conda activate slowdyn

$ conda install numpy

$ conda install scipy

$ conda install matplotlib

$ conda install scikit-learn

$ conda install jupyter notebook

$ conda install h5py

3. Download the slow dynamics protocol pipeline from the GitHub repository at https://github.com/shymkivy/slow_dynamics_protocol and extract it into a directory.

***Note:*** This pipeline contains the code required for all analyses described in this protocol, including binwise decoding, trial-to-trial variability analysis, and network INT analysis. Each method is accompanied by corresponding demo scripts.

### Data preparation

**Timing: <30 min**

**Figure 1:**
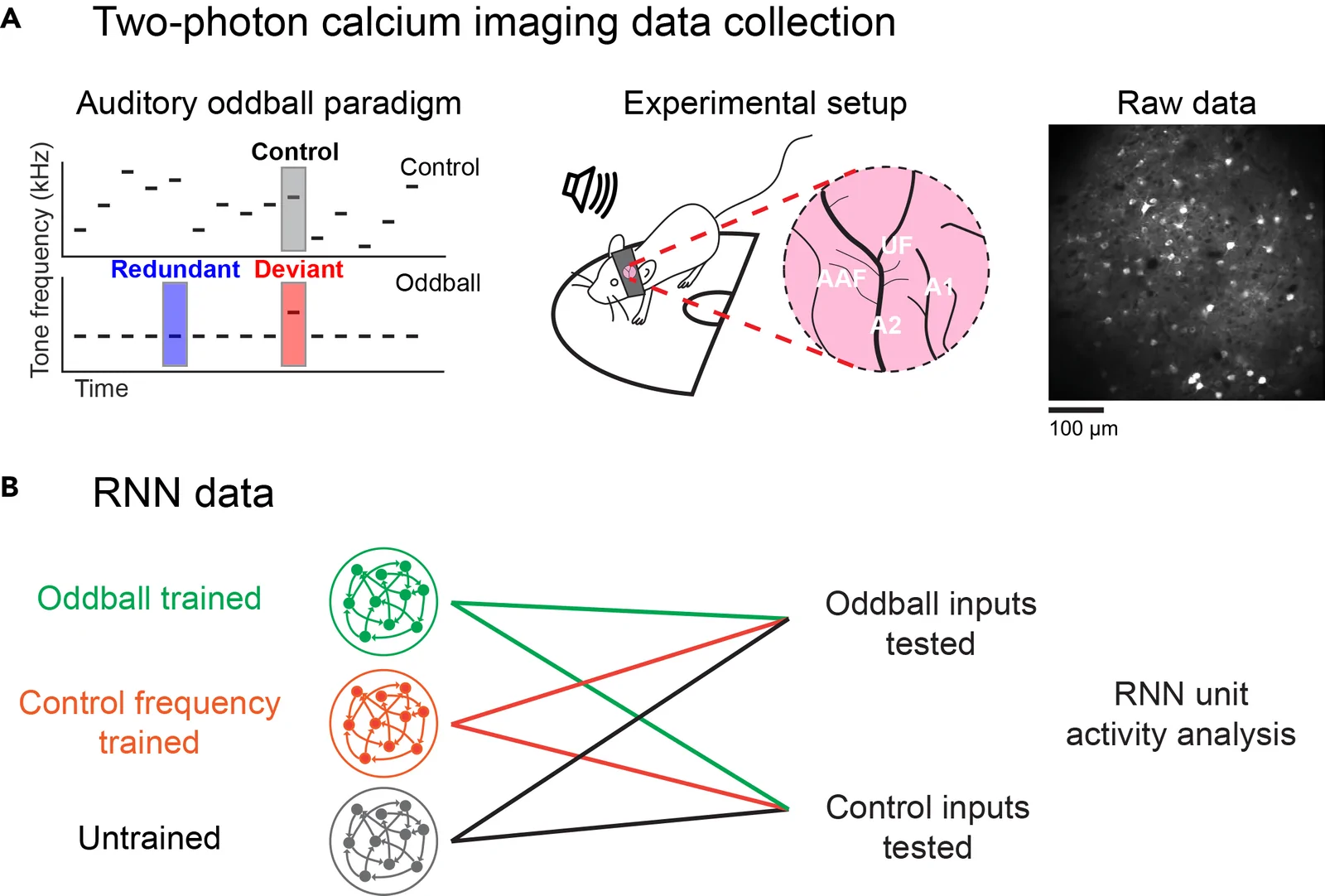
Data collection (A) Two-photon calcium imaging data were collected from awake mice during passive presentation of auditory stimuli in an oddball paradigm. (B) Artificial recurrent neural networks (RNNs) were trained using oddball or control inputs or were left untrained. The three network types were then tested with oddball and control inputs, and unit activity was extracted for analysis. Elements of panel (A) were reproduced from Shymkiv et al.^1^ Scale bar, 100 µm. A1, primary auditory cortex; A2, secondary auditory cortex; AAF, anterior auditory field; UF, ultrasonic field.

This protocol is intended for the analysis of time-series data from simultaneously recorded neurons. Neuronal activity may be represented as firing-rate traces, synaptic currents, or other suitable proxies. We first analyze two-photon calcium imaging datasets recorded from the auditory cortex of awake mice during passive presentation of auditory stimuli in an oddball paradigm (Figure 1A). Oddball stimuli elicit context-dependent activity, producing amplified neuronal responses to surprising events (novelty detection) and suppressed responses to redundant events (adaptation). Oddball-related processing is intrinsically temporal and requires short-term memory of recent stimuli, which may be encoded in the slow network dynamics analyzed here.

Second, we analyze neuronal activity from RNNs trained to perform novelty detection in an oddball task (oddball-trained), RNNs trained to perform sound-frequency recognition (control-trained), and untrained RNNs. After training, each network is tested with new oddball and control inputs drawn from the same stimulus distribution (Figure 1B). The activity of RNN units during the test phase is treated as analogous to calcium imaging data and analyzed using the methods described in this protocol.

The first two methods—binwise decoding and trial-to-trial variability analysis—are designed for control-like stimulus inputs, in which multiple stimulus types are presented to the mouse or network in a random sequence. The third method, an autocorrelation-based INT analysis adapted for neuronal networks, requires activity traces containing isolated activation events. Such events may occur during spontaneous activity or oddball stimulus presentation in calcium imaging data, or during oddball inputs in RNN-derived datasets. Table 1 lists the deposited datasets used in each analysis, together with the corresponding stimulus paradigm and required files.

4. Download the sample calcium imaging data from the data repository listed in the key resources table, unzip the downloaded file, and place its contents in a data directory.

***Note:*** These are population-level analyses; therefore, the numbers of simultaneously recorded neurons and trials should generally be maximized. The required sample size scales with the dimensionality of the neural activity representing the task. Because the analyses characterize population trajectories that reflect the underlying computation, a more complex task spans a higher-dimensional activity space and requires more neurons and trials for adequate sampling.^23^ The numbers of neurons and trials should be sufficient for the estimated dimensionality of the population activity to saturate and for estimates of trial-to-trial variability to stabilize. For a simple task—for example, one involving the presentation of 10 sensory stimuli—at least 10 neurons representing those stimuli are needed, although larger populations are preferable. As a practical guideline, we recommend recording a minimum of 10 neurons and repeating each stimulus at least 10 times for both the binwise decoding and trial-to-trial variability analyses. The intrinsic timescale (INT) analysis can instead be computed from spontaneous activity, for which a continuous recording is sufficient. When INT is computed from stimulus trials, sparsely spaced activations are preferable because they allow activity to decay between events. The exact requirements ultimately depend on task complexity and should be determined empirically.

5. Download the RNN test data from the repository listed in the key resources table. These data will be analyzed in parallel with the calcium imaging recordings.

***Note:*** Other datasets with simultaneous recordings of neuronal activity may also be used, although the data-loading steps must be modified to accommodate the alternative data format.

**Table 1:** Deposited datasets used by each analysis

Analysis	Data source	Input paradigm	What is analyzed	Deposited files
Binwise decoder	Calcium imaging	Control (10 frequencies)	Frequency identity	Oddball (“mismatch”), M1–M10
Binwise decoder	RNN	Control (50 frequencies)	Frequency identity	RNN ∗_cont_data.npy + ∗_params.npy
Binwise decoder	RNN	Oddball (all pairwise combinations of 50 frequencies)	Frequency identity	RNN ∗_ob_data.npy + ∗_params.npy
Trial-to-trial variability	Calcium imaging	Variable ISI control runs	Trial-to-trial correlation of identical stimuli	Variable-ISI (“echo”) – M226, M4264, M4265, M4266, M4371, M4372
Intrinsic timescale (INT)	Calcium imaging	Full continuous trace (control and/or oddball)	Neuronal and network timescale	Oddball (“mismatch”), M1–M10
Intrinsic timescale (INT)	RNN	Full continuous trace (control or oddball)	Neuronal and network timescale	RNN ∗_cont_data.npy (or ∗_ob_data.npy) + ∗_params.npy

including the compatible data source (calcium imaging or RNN), input stimulus paradigm, decoded or measured quantity, and required files from the Dryad repository.

## Key resources table

**Table:** 

REAGENT or RESOURCE	SOURCE	IDENTIFIER
**Deposited Data**		

Calcium imaging and RNN data	Shymkiv et al.^1^^,^^24^	Dryad Data: https://doi.org/10.5061/dryad.xsj3tx9q6

**Software and algorithms**		

Python 3 (v3.12.12)	Python Software Foundation	https://www.python.org; RRID:SCR_008394
Miniconda	Anaconda, Inc.	https://www.anaconda.com; RRID:SCR_018317
NumPy (v2.3.5)	Harris et al.^25^	https://numpy.org; RRID:SCR_008633
SciPy (v1.16.3)	Virtanen et al.^26^	https://scipy.org; RRID:SCR_008058
Scikit-learn (v1.7.1)	Pedregosa et al.^27^	https://scikit-learn.org; RRID:SCR_002577
Jupyter Notebook (v7.5.0)	Kluyver et al.^28^	https://jupyter.org; RRID:SCR_018315
Matplotlib (v3.10.7)	Hunter^29^	https://matplotlib.org; RRID:SCR_008624
h5py (v3.15.1)	Collette^30^	https://www.h5py.org; RRID:SCR_024812
Slow dynamics analysis pipeline	This paper	Github: https://github.com/shymkivy/slow_dynamics_protocol

## Step-by-step method details

This section outlines the steps for loading, processing, and analyzing slow dynamics in neuronal recordings. Calcium imaging datasets are analyzed in parallel with RNN unit activity.

### Data loading and preprocessing

**Timing: <5 min**

This section launches the analysis environment and loads the example datasets, converting the raw data into the firing-rate matrices, trial identities, and stimulus-onset times used by all subsequent analyses.

1. To run the pipeline, open the Anaconda Prompt on Windows or a terminal on Linux or macOS and launch Jupyter Notebook.

$ conda activate slowdyn

$ jupyter notebook

***Note:*** The pipeline can be run using the provided demo scripts (*.ipynb* or *.py*) included in the slow dynamics pipeline.

2. After opening a demo script or creating a new one, first import the required libraries.
  a. To import functions from the slow dynamics library, set the “pipeline_dir” variable to the directory containing the library.
    >pipeline_dir = ‘your analysis pipeline directory goes here’ # edit this
  b. Import all required libraries and functions.
    # ---- importing functions ----
    >import sys
    >import numpy as np
    >import matplotlib.pyplot as plt
    >from scipy.spatial.distance import pdist, squareform
    # importing slow dynamics pipeline
    >sys.path.append(pipeline_dir + '/functions')
    >import sd_utils as sd
    >import sd_decoder as dec
3. Set a fixed random seed to ensure that the results are reproducible across runs.

>seed = 0  # integer seed for reproducible results, None for random

**Figure 2:**
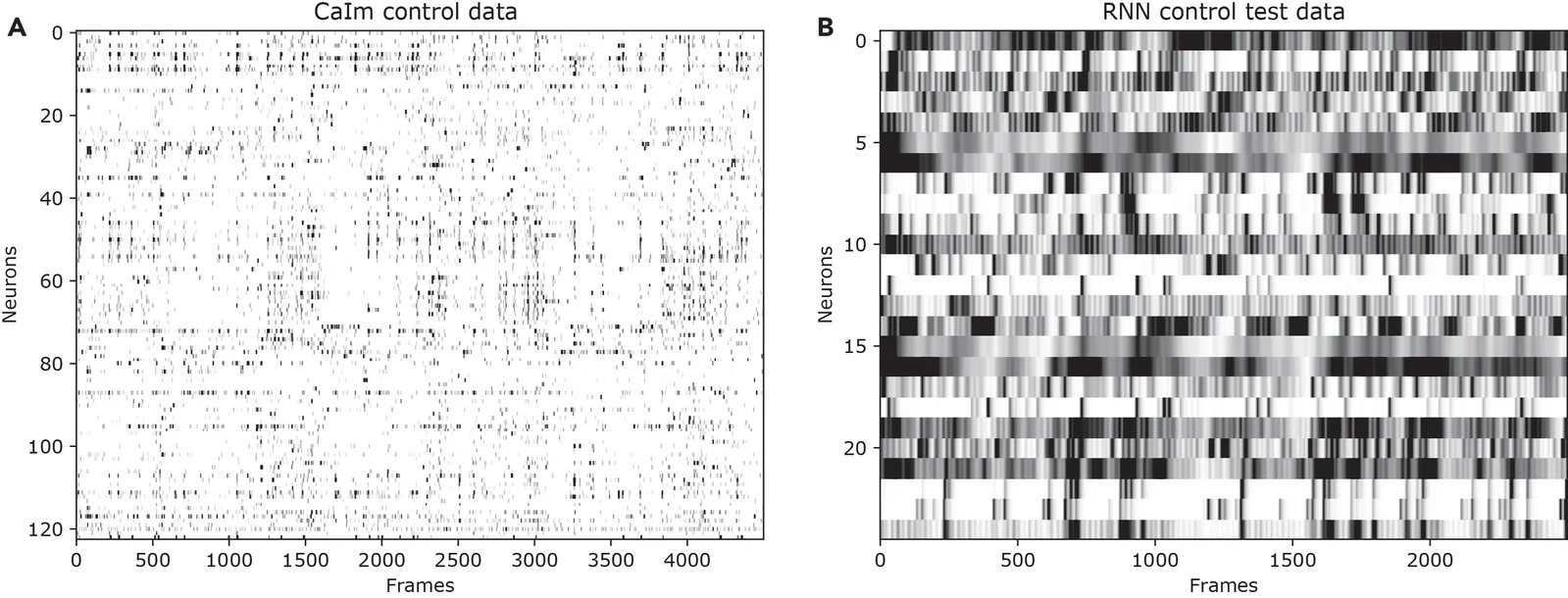
Example neuronal firing-rate rasters (A) Clcium imaging data. (B) RNN unit data.

4. Load and preprocess the calcium imaging (CaIm) data.
  a. Set the “data_dir” variable to the directory containing the sample data.
    >data_dir = ‘your CaIm data directory goes here’  # edit this
  b. Load the data.
    ***Optional:*** The number of datasets loaded can be limited using the “num_files” variable.
    i. Select the deconvolution algorithm used to estimate firing-rate traces; choose either “oasis” (CaImAn) or “smoothdfdt,” a smoothed, rectified first derivative.
      ***Note:*** The firing-rate traces can be smoothed using a Gaussian kernel specified by the “smooth_std_duration” variable (default standard deviation = 0.1 s).
      # ---- loading CaIm datasets ----
      # firing rates, trial types, and stimuli times
      >data_ob = sd.load_caim_data_mat(
        data_dir,                # data directory
        ext_list=['mat'],          # data extension
        tags=['ammn’, '_processed_data'],   # data tags
        num_files=20,              # limit number of files to load
        deconvolution='oasis’,           # deconvolution oasis or smoothdfdt
        smooth_std_duration=0.1)          # in seconds
      ***Note:*** The loading script returns the.mat data as a list of dictionaries (“data_ob”), one per recording. Each dictionary contains the firing-rate traces (“firing_rates,” a neuron × frame matrix), the stimulus-onset times (“stim_times”), and corresponding trial identities (“trial_types”). For external datasets, loading steps must be modified accordingly, and the data should be stored as a list of dictionaries representing individual recordings.
  c. Extract the frame rate used during calcium imaging for all datasets, or manually set the “frame_rate” variable when using external data.
    ***Note:*** The “frame_rate” variable is specified in Hz. The following code assumes that the frame rate is approximately constant across datasets of the same type.
    >frame_rate = 1000/np.mean(sd.get_values(data_ob, ‘volume_period')) # in Hz
5. In parallel, load the RNN unit-activity datasets generated from oddball- and control-trained networks tested with control inputs.
  a. Set the “data_dir_rnn” variable to the directory containing the RNN test data.
  b. Load the datasets from RNNs tested with control inputs as a list of dictionaries, each representing an individual network.
    # ---- loading RNN testing with control datasets ----
    >data_dir_rnn = ‘your RNN data directory goes here’ # edit this
    >fname_rnn = ‘RNN_test_data_2024_5_24_9h_42m2'.
    >data_rnn = sd.load_rnn_test(
      data_dir_rnn,
      fname_rnn + '_cont_data.npy',
      fname_rnn + '_params.npy',
      max_net_load = 5,
      flatten_runs = True,
      max_trial_types = 10,
      max_trials = 400,
      limit_network_types=['ob trained’, ‘freq trained'],
      seed=seed)
    ***Note:*** The structure of the RNN datasets matches that of the calcium imaging data, allowing reuse of the same analysis functions. The analyzed RNNs contain 25–50 units; in the original study, this range was sufficient to achieve optimal performance.
  c. Compute an equivalent frame rate (“frame_rate_rnn”) from the RNN time step of 0.05 s.
  d. Load the “training_type” array indicating whether each dataset originated from an oddball-trained, control-trained, or untrained network.
    >frame_rate_rnn = 1000/np.mean(sd.get_values(data_rnn, ‘volume_period'))
    >training_type = np.array(sd.get_values(data_rnn, ‘training'))
6. Plot example firing-rate rasters for calcium imaging data and RNN units (Figure 2).

# ---- plot example firing-rate raster ----

>fig = sd.plot_fig_raster(

  data_ob[0]['firing_rates'][:,500:5000],

  sd.normalize(data_rnn[0]['firing_rates'][:,2000:4500]),

  frame_rate=frame_rate,

  frame_rate_rnn=frame_rate_rnn)

7. In the final preprocessing step, reorganize the 2D firing-rate matrix (neurons × frames) into a 3D matrix containing single-trial response data (neurons × frames × trials).
  a. First, compute the number of frames to extract before stimulus onset (baseline) and after stimulus onset (stimulus response).
    ***Note:*** The trial window corresponds to the duration over which activity around each trial is analyzed. In the “trial_win” variable, “−1” corresponds to a 1 s baseline, and “3” corresponds to a 3 s response window. The frame rate must also be provided to convert time into frames.
    # ---- CaIm data extracting trials using stimulus times ----
    # compute trial window frames (assuming all datasets have similar frame rate)
    >trial_frames, plot_t = sd.get_frames(
      trial_win=[-1,3], # seconds relative to stim onset (pre, post)
      frame_rate=frame_rate)
  b. Loop over each dataset and extract the 3D stimulus-triggered response matrices, stored in the “stim_trig_resp_all” list.
    # extract stimulus-triggered average matrix (neurons, frames, trials)
    >stim_trig_resp_all = []
    >for n_fl in range(len(data_ob)):
    >  stim_trig_resp = sd.get_stim_trig_resp(
       data_ob[n_fl]['firing_rates'],  # firing rates
       data_ob[n_fl]['stim_times'],  # stimulus onset frames
       trial_frames=trial_frames)    # frames to be extracted
    >  stim_trig_resp_all.append(stim_trig_resp)
8. Similarly, extract stimulus-triggered response data for the RNN datasets and store them in the “stim_trig_resp_all_rnn” list.
  a. For the RNN data, use a (−1, 5) s window for the baseline and stimulus response, as the slow dynamics in these networks persist over longer timescales.

# ---- RNN tested with control inputs ----

# RNN compute trial window frames for stim-triggered responses

>trial_frames_rnn, plot_t_rnn = sd.get_frames(

  trial_win=[-1,5],   # seconds relative to stim onset (pre, post)

  frame_rate=frame_rate_rnn)

# computing stimulus-triggered average (neurons, frames, trials)

>stim_trig_resp_all_rnn = []

>for n_fl in range(len(data_rnn)):

>  stim_trig_resp = sd.get_stim_trig_resp(

   data_rnn[n_fl]['firing_rates'], # firing rates

   data_rnn[n_fl]['stim_times'], # stimulus onset frames

   trial_frames=trial_frames_rnn) # frames to be extracted

>  stim_trig_resp_all_rnn.append(stim_trig_resp)

### Binwise decoder

**Timing: <30 min**

This section describes the use of a binwise decoder to analyze slow network dynamics in calcium imaging and RNN data. Three-dimensional stimulus-triggered response matrices are used to decode the corresponding stimulus identities. In the binwise structure, a separate decoder is trained and tested at each time bin within the trial to determine the stimulus identity; this is also referred to here as the “diagonal” structure. Alternatively, a decoder trained at one time bin can be tested at all other time bins, producing a “full” structure. The diagonal structure reveals the duration over which stimulus-related activity persists in the network. The full structure indicates whether the neuronal population remains in the same network state or progresses along a trajectory corresponding to a sequence of stimulus-related neuronal activations. The binwise decoder can be run using the *slow_dynamics_decoder_comb_data_rnn.ipynb* demo script.

9. First, set up the “f_diag_decoder()” function to run the decoder in a diagonal binwise structure.
  ***Note:*** For each trial time bin, the binwise decoder is trained and then tested to determine the corresponding trial identity. A linear support vector machine (SVM) decoder is used to classify trial types in a one-vs-all setup, with five-fold cross-validation to estimate performance.
  a. Input the data to be decoded in the “X_all” variable and the trial identities (“trial_types”) in “Y_all.” For label shuffling, append a duplicate of the data in “X_all” together with the “trial_types” within the “shuffle_trials()” function.
  b. Specify parameters for running the diagonal decoder and five-fold cross-validation as inputs to the “run_binwise_dec()” function.
    ***Optional:*** The data can be processed with principal component analysis (PCA) and reduced to a specified fraction of the total variance, as determined by the “pca_var_frac” variable.
    # ---- Define diagonal binwise decoder ----
    >def f_diag_decoder(stim_trig_resp, trial_types, seed=None):
    >  X_all = [stim_trig_resp,         # data
         stim_trig_resp]       # data repeat for label-shuffled control
    >  Y_all = [trial_types,           # trial labels
        dec.shuffle_trials(trial_types, seed=seed)] # label-shuffling
    >  dec_data = dec.run_binwise_dec(
       X_all,
       Y_all,
       train_test_method='diag’,  # options: full, diag
       pca_var_frac=1,     # reduce data with pca
       num_cv=5,       # cross validation
       normalize=False,
       add_noise_sigma=1e-5,   # for stability
       get_train_coeffs=True,
       seed=seed)
    >  return dec_data
10. Train the diagonal binwise decoder in a loop over all datasets.

# ---- Run for all CaIm datasets ----

>dec_data_all = []

>for n_fl in range(len(data_ob)):

>  print(‘Training dataset %d/%d' % (n_fl+1, len(stim_trig_resp_all)))

>  trial_types = data_ob[n_fl]['trial_types']

>  trial_types_use = trial_types<10 # select trials to use

>  dec_data = f_diag_decoder(

   stim_trig_resp_all[n_fl][:,:,trial_types_use],

   trial_types[trial_types_use],

   seed=seed)

>  dec_data_all.append(dec_data)

>print(‘Done')

11. Similarly, train the diagonal binwise decoder on all RNN test datasets.

# ---- Run for all RNN datasets ----

>dec_data_all_rnn = []

>for n_fl in range(len(data_rnn)):

>  print(‘Training dataset %d/%d' % (n_fl+1, len(stim_trig_resp_all_rnn)))

>  dec_data = f_diag_decoder(

   stim_trig_resp_all_rnn[n_fl],

   data_rnn[n_fl]['trial_types'],

   seed=seed)

>  dec_data_all_rnn.append(dec_data)

>print(‘Done')

**Figure 3:**
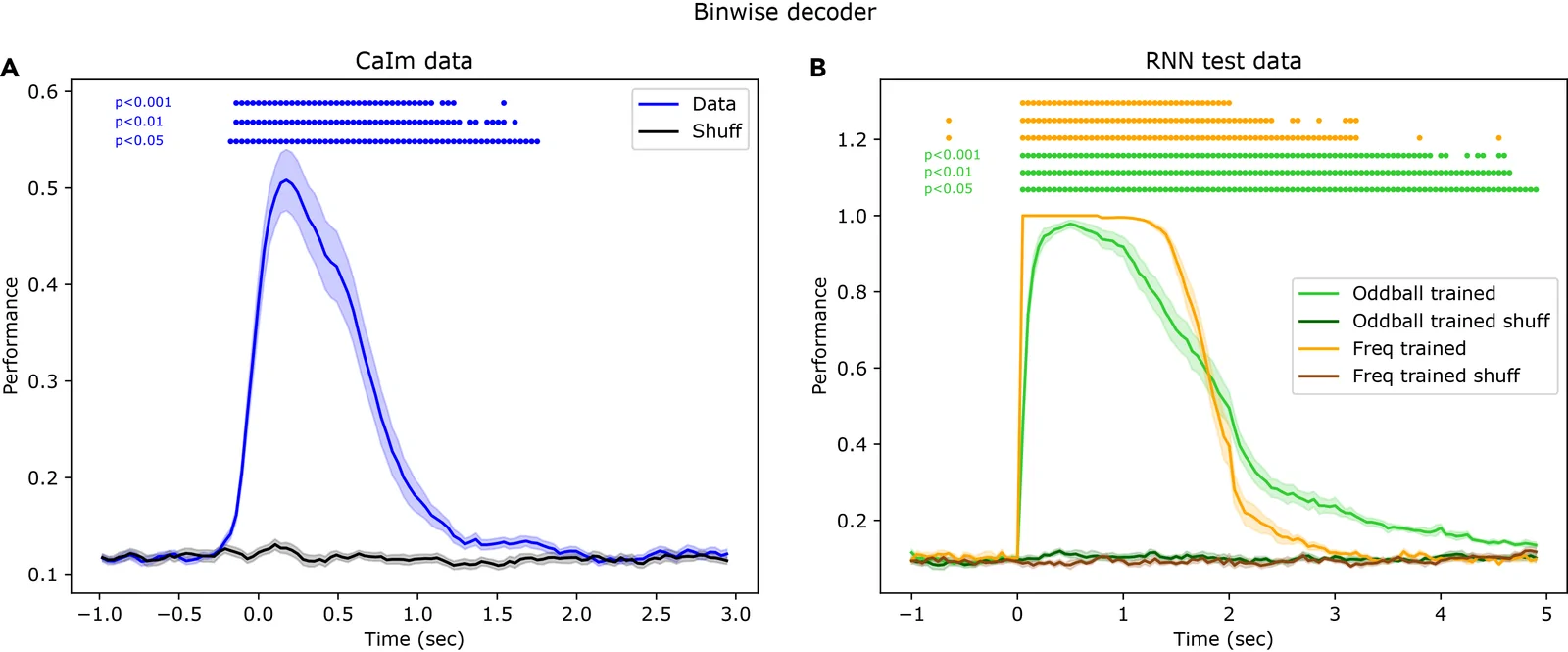
Diagonal binwise decoding Decoding reveals the persistence of stimulus-related activity in (A) calcium imaging datasets and (B) data from RNNs tested with control inputs. Stimuli were presented from 0 to 0.5 s, followed by a 0.5 s inter-stimulus interval (ISI). Traces show the mean across datasets; shading indicates ±SEM. Performance is the fraction of correctly classified single trials. Rows of markers above the traces indicate time bins at which decoding exceeded the label-shuffled control (two-sample t test; from bottom to top, p < 0.05, p < 0.01, and p < 0.001).

12. Plot the diagonal decoder results (Figure 3).

# ---- Plotting diagonal decoder results ----

>sd.plot_fig_diag_decoder(

  dec_data_all,

  dec_data_all_rnn,

  training_type,

  plot_t=plot_t,

  plot_t_rnn=plot_t_rnn)

***Note:*** The data reveal that stimulus-related activity persists beyond the stimulus duration (0–0.5 s; Figure 3). Activity trajectories in oddball-trained RNNs persist longer than those in control-trained RNNs, indicating that oddball computation in RNNs requires information to be stored over time.

13. To reveal whether the network maintains the slow dynamics in a single state or transitions along a trajectory, run a full binwise decoder.

***Note:*** This approach, also known as cross-temporal or temporal-generalization decoding,^16^^,^^17^ tests whether the population code is static (sustained: a decoder trained at one time bin generalizes across other time bins) or dynamic (evolving along a trajectory: decoding remains time-specific).

# ---- Define full decoding space binwise decoder ----

>def f_full_decoder(stim_trig_resp, trial_types, seed=None):

>  X_all = [stim_trig_resp,        # data

       stim_trig_resp]       # data repeat for label-shuffled control

>  Y_all = [trial_types,           # trial labels

    dec.shuffle_trials(trial_types, seed=seed)]  # label-shuffling

>  dec_data = dec.run_binwise_dec(

   X_all,

   Y_all,

   train_test_method='full’,  # options: full, diag

   pca_var_frac=1,    # reduce data with pca

   num_cv=5,       # cross validation

   normalize=False,

   add_noise_sigma=1e-5,   # for stability

   log=True,

   seed=seed)

>  return dec_data

***Note:*** As with the diagonal binwise decoder, each dataset is decoded alongside a label-shuffled version.

14. Run the decoder for an example calcium imaging dataset.

# ---- Run example CaIm dataset ----

>n_fl = 0   # select dataset

>trial_types = data_ob[n_fl]['trial_types']

>trial_types_use = trial_types<10 # select trials to use

>dec_data_full = f_full_decoder(

   stim_trig_resp_all[n_fl][:,:,trial_types_use],

   trial_types[trial_types_use],

   seed=seed)

**Figure 4:**
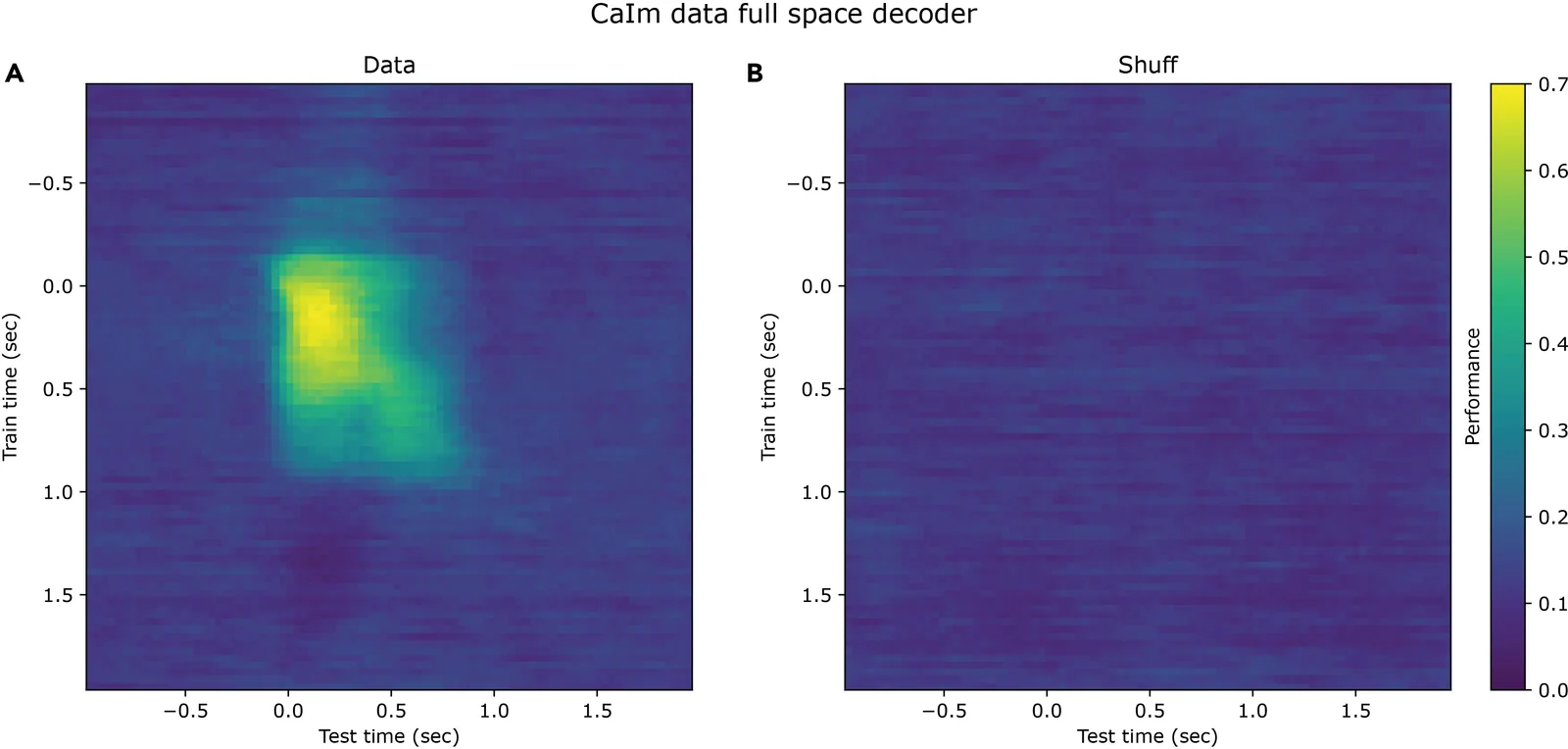
Full binwise decoding of calcium imaging data (A) Data and (B) label-shuffled control. Differences in decoding performance across training and testing time points indicate the evolution of population activity over time.

15. Plot the full decoder for the example calcium imaging dataset (Figure 4).

# ---- Plotting full decoder results CaIm data ----

>fig_full = sd.plot_fig_full_decoder_caim(

  dec_data_full, plot_t,

  clim=[0, 0.7])

***Note:*** The data reveal that decoding profiles change across decoder-training time bins, indicating the evolution of an activity trajectory (Figure 4).

16. Run the full binwise decoder for an example RNN test dataset.

# ---- Run example RNN datasets ----

# oddball-trained control-tested

>n_fl = np.where(training_type == ‘ob trained')[0][0]

>dec_data_full_ob = f_full_decoder(

  stim_trig_resp_all_rnn[n_fl],

  data_rnn[n_fl]['trial_types'])

# control-trained control-tested

>n_fl = np.where(training_type == ‘freq trained')[0][0]

>dec_data_full_freq = f_full_decoder(

  stim_trig_resp_all_rnn[n_fl],

  data_rnn[n_fl]['trial_types'])

**Figure 5:**
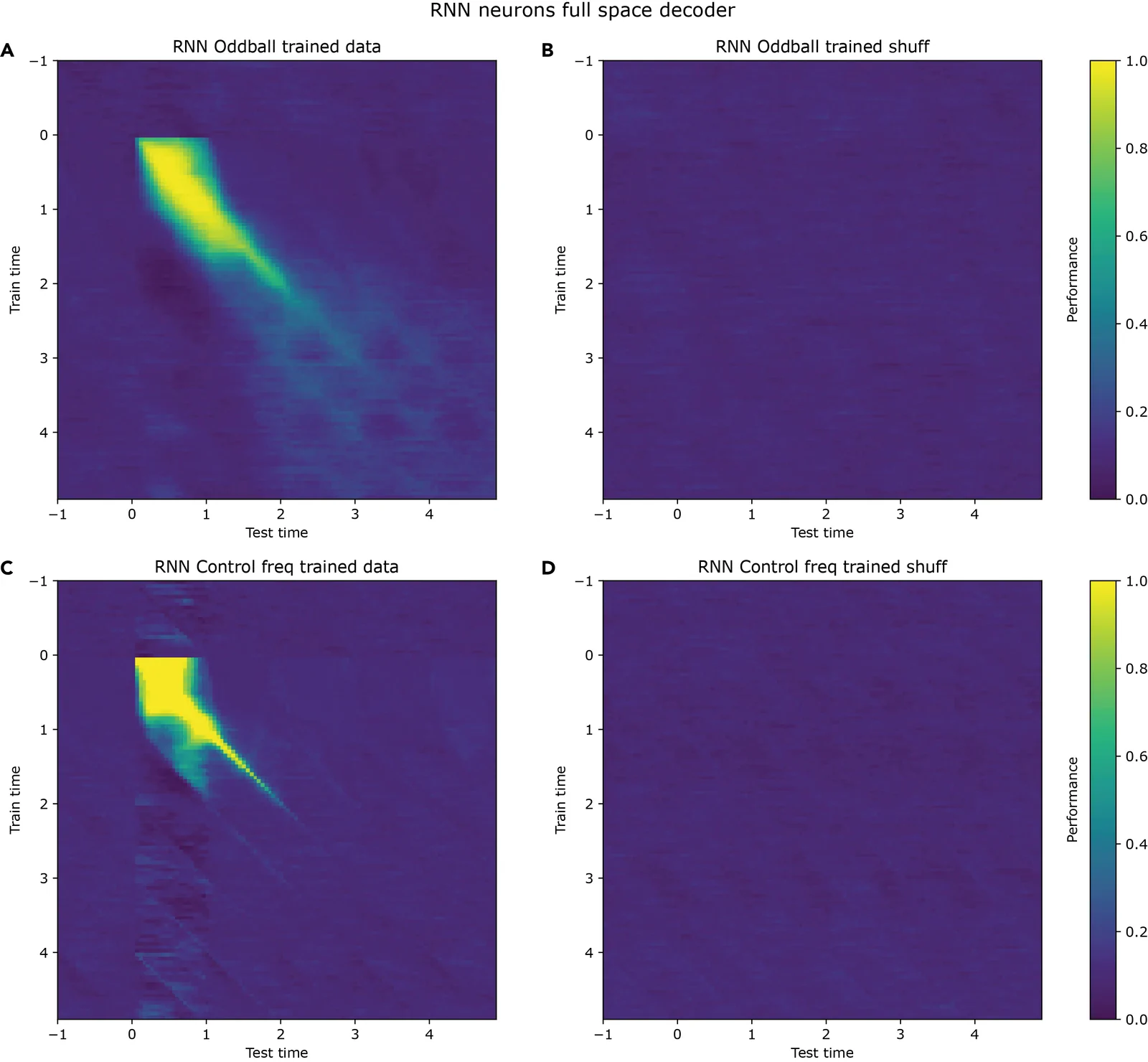
Both control- and oddball-trained RNNs show movement of the population state along a trajectory during the stimulus response Decoding results are shown for (A) an oddball-trained RNN, (B) its label-shuffled control, (C) a control-trained RNN, and (D) its label-shuffled control.

17. Plot the full decoder in decoding space (Figure 5).

# ---- Plotting full decoder results RNN data ----

>sd.plot_fig_full_decoder_RNN(

  dec_data_full_ob,

  dec_data_full_freq,

  plot_t_rnn)

***Note:*** The plot reveals a trajectory of activity in response to stimuli, as opposed to a single sustained response state (Figure 5).

### Trial-to-trial variability analysis

**Timing: <5 min**

This section analyzes the effects of slow network dynamics on trial-to-trial variability during control runs of the oddball paradigm. The standard oddball paradigm and its control condition use relatively short inter-stimulus interval (ISI; 0.5 s). Because novelty detection requires tracking recent stimuli, the response to each incoming stimulus is expected to be influenced by persistent activity elicited by preceding stimuli, resulting in increased trial-to-trial variability. Increasing the ISI is expected to reduce this variability.

This section analyzes extended control datasets with four ISIs: 0.5, 1, 2, and 4 s. The trial-to-trial correlation analysis described here can be performed using the *slow_dynamics_isi_correlation.ipynb* demo script.

18. Load calcium imaging data from control runs with variable ISIs (0.5, 1, 2, and 4 s).
  a. Provide the directory in the “data_dir” variable.
  b. Specify the frame rate variable.
  c. Extract an array of ISI values corresponding to the loaded datasets.

## ---- loading echo data ----

>data_dir = ‘your variable ISI data directory goes here’ # edit this

# loading raw firing rates, trial types, and stimuli times from echo dataset

>data_echo = sd.load_caim_data_mat(

  data_dir,           # data directory

  ext_list=['mat'],        # data extension

  tags=['cont’, '_processed_data'],   # data tags

  num_files=None,       # limit number of files to load

  deconvolution='smoothdfdt’,     # deconvolution oasis or smoothdfdt

  smooth_std_duration=0.15)     # in seconds

>frame_rate = 1000/np.mean(sd.get_values(data_echo, ‘volume_period')) # Hz

>isi_list = np.array(sd.get_values(data_echo, ‘isi'))

***Note:*** The datasets are loaded in the same format as in previous sections. The “data_echo” variable is a list of dictionaries representing individual recordings, each containing keys for firing-rate traces, stimulus-onset times, and trial types.

**Figure 6:**
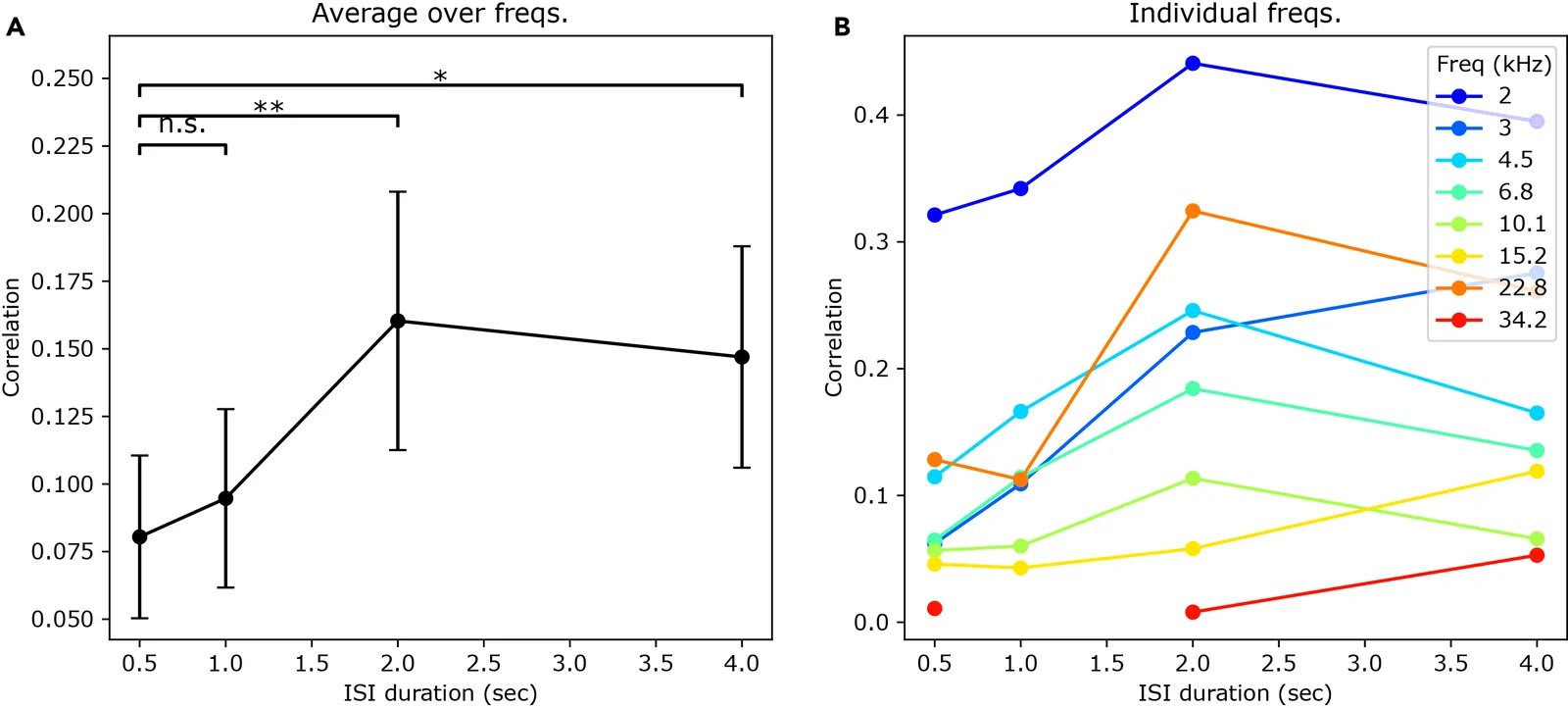
Trial-to-trial correlation increases as the inter-stimulus interval (ISI) increases; equivalently, trial-to-trial variability is highest at the shortest ISI and decreases at longer ISIs Results are shown across all frequencies (A) and for individual frequencies (B). Points show the mean across datasets; error bars indicate ±SEM. In (A), each longer ISI was compared with the 0.5 s ISI using a two-sided Mann–Whitney U test (∗ p < 0.05, ∗∗ p < 0.01, ∗∗∗ p < 0.001).

19. Compute stimulus-response statistics and identify responsive cells.
  a. First, specify the trial window over which the 3D stimulus-triggered response matrix will be extracted for computing response statistics (−1 s to 2 s relative to stimulus onset).
    # compute the frames
    >trial_frames_tuning, plot_t_tuning = sd.get_frames(
      trial_win=[-1, 2], # seconds relative to stim onset (pre, post)
      frame_rate=frame_rate)
    ***Note:*** An extended window is used to estimate stimulus-response statistics to ensure that delayed activity is also captured.
  b. Next, for each dataset, extract the stimulus-triggered average and use it to compute stimulus tuning for each cell.
  c. Extract a Boolean array indicating which cells are responsive.
    # compute cell tuning and generate a list of responsive cells
    >resp_cells_all = []
    >for n_fl in range(len(data_echo)):
      # computing stimulus-triggered average (neurons, frames, trials)
    >  stim_trig_resp = sd.get_stim_trig_resp(
       data_echo[n_fl]['firing_rates'],   # firing rates
       data_echo[n_fl]['stim_times'],   # stimulus onset frames
       trial_frames=trial_frames_tuning)   # frames to be extracted
    >  print(‘computing stats dset %d/%d' % (n_fl+1, len(data_echo)))
    >  resp_cells = sd.compute_tuning(
       stim_trig_resp,
       data_echo[n_fl]['trial_types'],
       np.arange(1,11),
       plot_t_tuning,
       num_samp=2000,
       z_thresh = 3,
       sig_resp_win = [0, 1.2],
       seed=seed)
    >  resp_cells_all.append(resp_cells)
    ***Note:*** Responsive cells are identified using a bootstrap-based peak-detection method. For each cell and stimulus type, the stimulus-triggered response is first extracted over a trial window spanning −1 to 2 s around stimulus onset and averaged across trials. The peak of this trial-averaged response is then located, and its amplitude is taken as the mean activity over a short peak window (∼0.25 s) centered on that maximum. A null distribution of peak amplitudes is generated for each cell by repeating this measurement over 2,000 randomly drawn trial sets. Each set contains the same number of trials as the corresponding stimulus condition but is sampled irrespective of stimulus identity. A cell is labeled responsive to a given stimulus if its measured peak exceeds the null-distribution threshold corresponding to z = 3 and occurs within the 0–1.2 s post-stimulus response window. Other statistical approaches for identifying responsive cells may also be used.
20. For each dataset, extract activity from trials with identical stimulus identities and compute trial-to-trial correlations.
  a. First, define the trial time window over which to compute the correlations. The window (−0.05, 0.95) corresponds to −0.05 to 0.95 s relative to stimulus onset and is appropriate for a paradigm with a 0.5 s stimulus duration and a 0.5 s ISI.
    ***Note:*** The trial window should not overlap substantially with surrounding trials, as this could introduce activity associated with adjacent stimuli.
  b. Generate a second version of stimulus-triggered response data organized by unique trial types (frequencies 1–10).
  c. Compute pairwise correlations for each trial type using Pearson’s correlation coefficient.
  d. For each unique frequency, compute a trial-to-trial correlation matrix and extract the mean correlation value, using only groups containing a sufficient number of responsive cells (default “min_resp_cells” = 5).
    ## ---- correlation analysis CaIm echo data ----
    >trial_frames, plot_t = sd.get_frames(
      trial_win = [-0.05, .95],   # seconds relative to stim
      frame_rate = frame_rate)
    >corr_vals_all = []
    >stim_trig_resp_all = []
    >for n_fl in range(len(data_echo)):
    >  stim_trig_resp = sd.get_stim_trig_resp(
       data_echo[n_fl]['firing_rates'],
       data_echo[n_fl]['stim_times'],
       trial_frames=trial_frames)
    >  stim_trig_resp_all.append(stim_trig_resp)
    >  corr_vals = sd.compute_correlation(
       stim_trig_resp,     # stimulus-triggered responses
       data_echo[n_fl]['trial_types'],  # vector of trial types
       np.arange(1,11),      # trials types to analyze (1-10)
       resp_cells = resp_cells_all[n_fl],  # responsive cells array
       metric='correlation’,     # cosine, correlation
       min_resp_cells=5,    # minimum responsive cells
       seed=seed)
    >  corr_vals_all.append(corr_vals)
    >corr_vals = np.vstack(corr_vals_all)
21. Plot the computed correlation values as a function of ISI (Figure 6).

**Figure 7:**
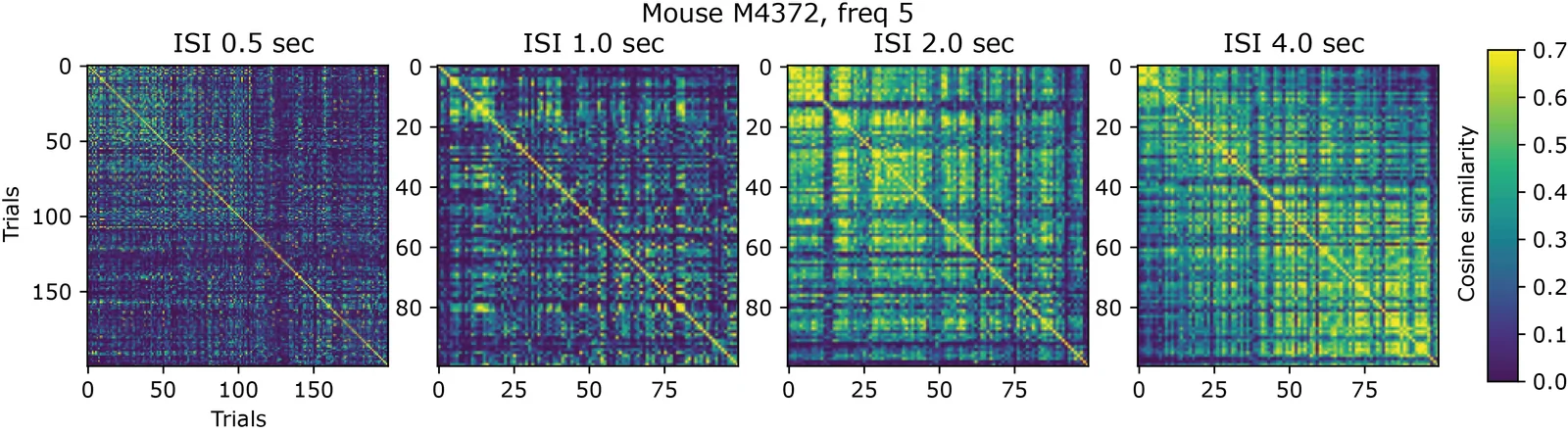
Examples of trial-to-trial cosine-similarity matrices at different ISIs Panels show, from left to right, ISIs of 0.5, 1, 2, and 4 s for one example dataset. Axes are trial index; colour indicates the cosine similarity between the population response vectors of each pair of trials.

# ---- plot correlation for trials 1-10 across ISI ----

>fig, ax, groups = sd.plot_fig_isi_corr_trials(

  corr_vals,

  isi_list,

  colormap='jet',

  metric_tag = ‘Correlation')

# statistical comparison:

>sd.stat_compare(ax[0], groups, ‘ISI 0.5′, ‘ISI 1′, test='mannwhitney',

 alternative='two-sided')

>sd.stat_compare(ax[0], groups, ‘ISI 0.5′, ‘ISI 2′, test='mannwhitney',

 alternative='two-sided')

>sd.stat_compare(ax[0], groups, ‘ISI 0.5′, ‘ISI 4′, test='mannwhitney',

 alternative='two-sided')

22. Visualize the changes in trial-to-trial variability by plotting the trial-to-trial cosine similarity matrices for datasets with different ISIs.
  a. First, specify the example mouse and trial type to display (frequency 5).
  b. For each ISI, compute the cosine similarity matrix for the selected trial type.
    ## ---- example trial-to-trial cosine similarity ----
    >mouse_list, mouse_tag = sd.get_example_mouse(data_echo,
      example_mouse='M4372′)
    >n_freq = 5
    >isi_uq = np.unique(isi_list)
    >SI_list = []
    >for n_isi in range(len(isi_uq)):
    >  mouse_idx2 = np.where((mouse_list == mouse_tag)
        & (isi_list == isi_uq[n_isi]))[0][0]
    >  trial_idx = data_echo[mouse_idx2]['trial_types'] == n_freq
    >  stim_trig_resp = stim_trig_resp_all[mouse_idx2][:,:,trial_idx]
    >  SI = sd.compute_correlation_mat(
       stim_trig_resp,
       subtract_mean=True,
       add_noise_sigma=1e-5,
       metric='cosine',
       seed=seed)
    >  SI_list.append(SI)
  c. Plot the matrices (Figure 7).
    # ---- plot figure ----
    >fig = sd.plot_fig_SI_mat(
      SI_list,
      isi_uq,
      title_tag = ‘mouse %s, freq %d' % (mouse_tag, n_freq))
    ***Note:*** Increased trial-to-trial similarity is indicated by brighter colors, corresponding to higher cosine similarity and lower variability (Figure 7).

### Intrinsic timescale analysis

**Timing: <5 min**

The final section uses intrinsic timescale (INT) analysis to compare the duration of activations in networks and individual neurons. Historically, INT has predominantly been used to measure neuronal timescales, enabling comparisons across cortical regions^20^ and different disease states.^21^ Because neuronal INT is computed using the autocorrelation of a firing-rate trace, followed by estimation of the decay time constant, no stimulus information is required. Additionally, both spontaneous and stimulus-evoked neuronal activity can be used.

The INT analysis is extended here to the neural network level. To generate a proxy for network activity, a baseline activity vector is computed, and the activity at each time point is measured using the Euclidean distance from this baseline. This provides a population-level analog of the single-neuron INT: whereas the neuronal INT is computed from the autocorrelation of one neuron’s activity, the network INT is computed from the autocorrelation of a single scalar—the magnitude of the population’s deviation from its resting state. By design, it measures the duration over which the network remains displaced from baseline and is deliberately agnostic to whether that displacement reflects a single sustained population state or a sequence of distinct states; the binwise decoders resolve which of these underlies the dynamics. The results show that although single-neuron INTs remain short, the network INT can be longer (see expected outcomes).

The INT analysis can be performed using the *slow_dynamics_net_int.ipynb* demo script.

**Figure 8:**
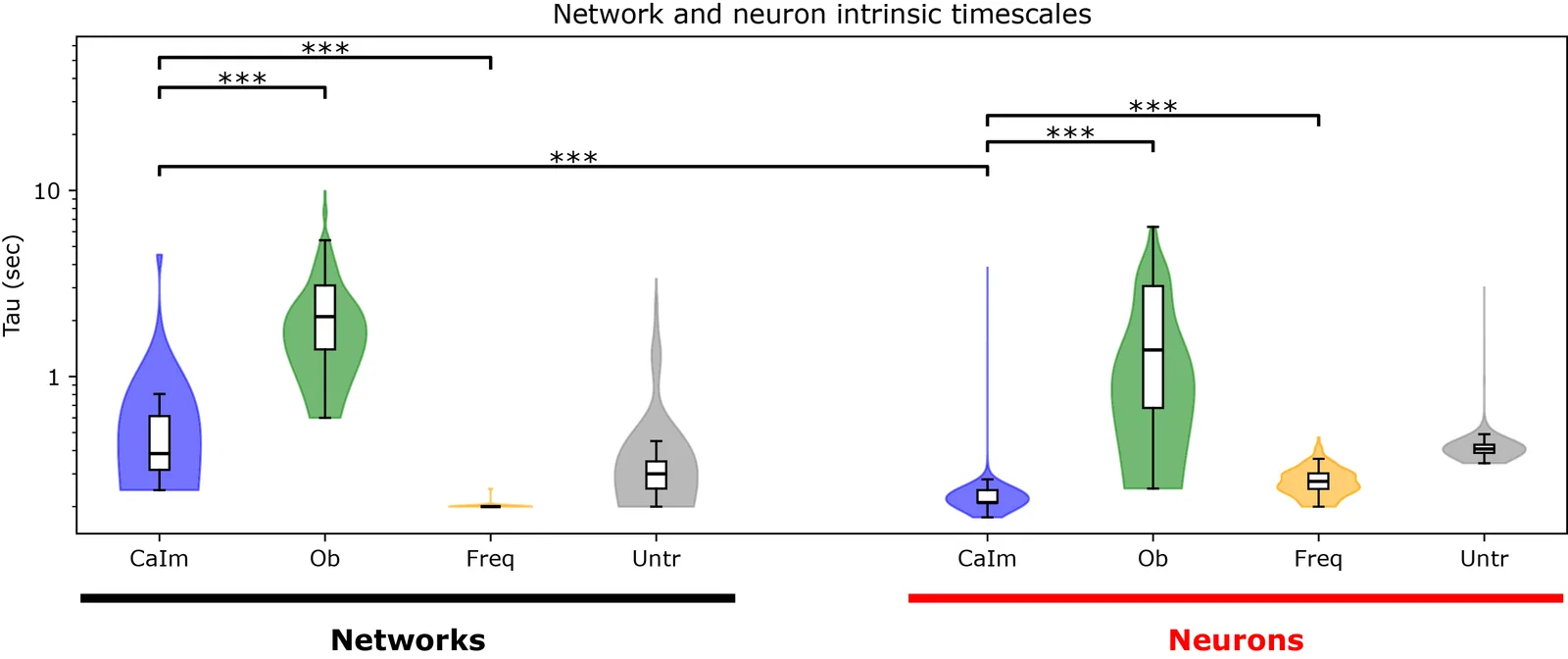
Intrinsic timescales (INT) plots for calcium imaging and RNN test data showing values for network timescales (left) and neuronal timescales (right) Violins show the distribution of tau across networks (left) or neurons (right); inset boxes show the median and interquartile range. Brackets indicate two-sided Mann–Whitney U test (∗∗∗ p < 0.001).

23. Load the data.
  a. Load the calcium imaging datasets using the code from steps 2–4.
    ***Note:*** For INT analysis, calcium imaging data are not organized into trials, unlike in previous sections, since the analysis does not require stimulus timing information.
  b. Reload the RNN data using parameters modified from those in the previous sections.
    # ---- loading RNN neuronal activity during oddball or control inputs ----
    >data_dir_rnn = ‘your RNN data directory goes here’ # edit this
    >fname_rnn = ‘RNN_test_data_2024_5_24_9h_42m2'.
    >data_rnn = sd.load_rnn_test(
      data_dir_rnn,
      fname_rnn + '_ob_data.npy',
      fname_rnn + '_params.npy',
      max_net_load = 5,
      flatten_runs = False,
      cut_zero_trials = True,
      num_initial_trials_skip = 20,
      limit_network_types=['ob trained’, ‘freq trained’, ‘untrained'],
      seed=seed)
    >frame_rate_rnn = 1000/np.mean(sd.get_values(data_rnn, ‘volume_period'))
    >training_type = np.array(sd.get_values(data_rnn, ‘training'))
    ***Note:*** Three network types are loaded: oddball-trained, control-trained, and untrained. The firing rate data here correspond to tests with oddball inputs, but control input data can be used as well. When the “flatten_runs” parameter is set to False, the firing rate data are loaded as a 3D matrix (runs × neurons × time). The “num_initial_trials_skip” parameter discards the first 20 RNN trials to avoid potential effects of large network transitions that may occur at the beginning of the input sequence.
24. Compute INT values for networks and neurons across all calcium imaging and RNN datasets using the “get_network_intrinsic_timescales()” function.
  # ---- Compute intrinsic timescales for CaIm data ----
  >tau_ob_net_all = []
  >tau_ob_cell_all = []
  >for n_dset in range(len(data_ob)):
  >  print(‘CaIm dset %d' %(n_dset))
  >  tau_net, tau_cell = sd.get_network_intrinsic_timescales(
     data_ob[n_dset]['firing_rates'],
     frame_rate)
  >  tau_ob_net_all.append(tau_net)
  >  tau_ob_cell_all.append(tau_cell)
  ***Note:*** For individual neurons, INT is estimated by first computing the autocorrelation of each neuronal firing rate trace. One approach is to fit the autocorrelation with an exponential decay function to estimate the decay time constant. In our analysis, we found that a more stable method is to identify the first time point, moving outward from zero lag, at which the autocorrelation falls below half of its maximum value.^31^ This method is more stable for signals with dense activations.
  ***Note:*** To analyze INT at the network level, we first estimate a network activity trace and then apply the same single-neuron INT procedure. The network activity trace is the Euclidean distance between the instantaneous population vector and a baseline vector: the baseline vector, b, is the mean activity of each neuron across the entire recording (b = ⟨r(t)⟩_t_), where r(t) is the N-neuron activity vector at frame t, and the trace is d(t) = ‖r(t) − b‖_2_. The intrinsic timescale is then estimated from the autocorrelation of d(t). The baseline used here is the global mean activity across the recording. Because the analysis uses deconvolved calcium data, the signal is largely free of baseline drift; other datasets may require detrending prior to baseline computation. Firing rates are also normalized for each neuron so that no single neuron dominates d(t). Because INT is derived from the autocorrelation of d(t), it is insensitive to a constant baseline offset.
  a. First, run the network and neuronal INT analyses on all calcium imaging datasets.
  b. Next, run the same analyses on all RNN datasets.
    # ---- Compute intrinsic timescales for RNN data ----
    >tau_rnn_net_all = []
    >tau_rnn_cell_all = []
    >for n_rnn in range(len(data_rnn)):
    >  print(‘rnn %d' %(n_rnn))
    >  tau_net, tau_cell = sd.get_network_intrinsic_timescales(
       data_rnn[n_rnn]['firing_rates'],
       frame_rate_rnn)
    >  tau_rnn_net_all.append(tau_net)
    >  tau_rnn_cell_all.append(tau_cell)
25. Plot the INT values (“tau”) for both networks and neurons (Figure 8).

# ---- Plotting intrinsic timescales for CaIm and RNN data ----

>fig, ax, groups = sd.plot_fig_tau_networks_comb(

  tau_ob_net_all,

  tau_rnn_net_all,

  training_type,

  tau_ob_cell_all,

  tau_rnn_cell_all,

  do_log=True)

# statistical comparison:

>sd.stat_compare(ax, groups, ‘CaIm net’, ‘CaIm neuron',

  test='mannwhitney’, alternative='two-sided')

>sd.stat_compare(ax, groups, ‘CaIm neuron’, ‘Ob neuron',

  test='mannwhitney’, alternative='two-sided')

>sd.stat_compare(ax, groups, ‘CaIm neuron’, ‘Freq neuron',

  test='mannwhitney’, alternative='two-sided')

>sd.stat_compare(ax, groups, ‘CaIm net’, ‘Ob net',

  test='mannwhitney’, alternative='two-sided')

>sd.stat_compare(ax, groups, ‘CaIm net’, ‘Freq net',

  test='mannwhitney’, alternative='two-sided')

***Note:*** In the auditory cortex, the network time constant is longer than the neuronal time constant, consistent with the idea that populations in early sensory cortex evolve along population trajectories (Figure 8). The network time constant can also be compared with the RNN network time constants, to the extent that both systems are driven by the same input stimuli. The control task does not require memory and therefore provides a lower reference for the range of time constants, whereas oddball-trained RNNs, optimized to retain stimulus history, provide an upper reference. The cortical network time constant falls between these references, consistent with the intermediate memory demands in a general-purpose cortical network.

## Expected outcomes

This analysis protocol presents three approaches for examining slow dynamics in time-series data from simultaneously recorded neurons. These analyses require only appropriately formatted activity data and are not specific to a brain area or species; in the original study, they were applied to data from both primary and secondary auditory cortex. The decoder-based and trial-to-trial variability analyses focus on stimulus-triggered responses, whereas the INT-based analysis can be applied to general activity traces.

The goal of these analyses is to identify evidence of prolonged network activity (Figures 3, 4, 6, and 8), which may support short-term memory and contribute to temporal computations such as novelty detection.

## Limitations

Decoders, as implemented in this protocol, can reveal evidence of slow dynamics and help determine whether these dynamics are expressed as a single sustained population response or as sequential activations of individual neurons. However, these methods provide an indirect readout of network dynamics and do not identify the specific neuronal circuits or mechanisms underlying decoder performance. In addition, successful decoding does not necessarily imply that the decoded information is explicitly read out or functionally used by the network.

Intrinsic timescale (INT) analysis relies on estimating autocorrelation decay and is therefore sensitive to data quality. Datasets with dense, highly correlated activity may produce unstable estimates of time constants, a problem we sought to mitigate in this protocol. In addition, the network-level INT metric relies on a reduced representation of population activity, measured as the Euclidean distance from a baseline state. This transformation treats distinct population states that are equidistant from baseline as equivalent. This reduction is intentional: network INT quantifies the duration for which population activity remains displaced from baseline rather than the identity or direction of the population state, which are instead characterized using the binwise decoders.

Analysis of temporal dynamics using two-photon calcium imaging is limited by the effective temporal resolution of calcium signals and the microscope frame rate (∼60 Hz). Calcium indicator signals provide an indirect and temporally filtered measure of neuronal firing. Deconvolution and associated processing steps may introduce smoothing, further affecting analyses of temporal dynamics. Therefore, these experiments and analyses should ideally be replicated using recordings with higher effective temporal resolution.

The biological validation presented here is limited to mouse auditory cortex. Although the analyses are area-agnostic, their application to other cortical regions or species may require adjusting parameters to account for differences in temporal resolution and activity statistics.

Comparisons between biological recordings and RNN activity should be interpreted with caution. The RNN architectures, training objectives, and input statistics may not fully capture the complexity of biological circuits. Accordingly, the timescales shown in Figure 8 should be interpreted as a functional comparison based on the stimulus structure shared by both systems, rather than as a literal correspondence between the RNN’s discrete time steps and biological seconds.

## Troubleshooting

### Problem 1

The binwise decoder runs slowly or fails to converge (related to steps 9–14).

### Potential solution

There are several reasons why the decoder may fail to converge.

• First, standardize or normalize the data. For normalization, set each neuron’s activity to have a baseline of zero and a maximum of one. Alternatively, standardize each neuron by subtracting the mean and dividing by the standard deviation.
• The dataset may be too large. You can reduce the dimensionality using principal component analysis (PCA) and retain only the components that explain approximately 70% of the total variance. This can be implemented with the “pca_var_frac” parameter, set to 0.7.
• The one-vs-all classification scheme may be too complex if there are many trial types. Consider simplifying the problem by reducing the number of trial types to two.

### Problem 2

Trial-to-trial variability does not show clear structure or differences (related to steps 20–21).

### Potential solution

Analysis of trial-to-trial variability across repeated presentations of identical stimuli can reveal distinct cortical states that the brain visits; however, this requires a sufficiently large number of trials. Experiments should be designed to maximize the number of trials collected, ideally until the estimated dimensionality or explained variance reaches a plateau. One way to estimate the required number of trials is to apply PCA and examine how the explained variance changes as more trials are added. The explained variance should eventually reach a plateau, indicating sufficient sampling.

### Problem 3

INT analysis produces spurious timescales that are too long or too short and are not physiologically meaningful (related to step 24).

### Potential solution

The INT method can be sensitive to the types of firing-rate traces used. A major source of error arises from estimating the decay time constant of firing-rate autocorrelation. Ideally, traces should be mostly quiescent and contain sparse activations. For this reason, INT is often computed using spontaneous activity in animal models.

In datasets with dense activations, the autocorrelation may be difficult to interpret. To troubleshoot this issue, the user should plot the autocorrelation trace and assess whether the decay time constant can be reliably estimated. If not, the algorithm may need to be adjusted accordingly.

## Resource availability

### Lead contact

Requests for further information and resources should be directed to and will be fulfilled by the lead contact, Yuriy Shymkiv (ys2605@columbia.edu).

### Technical contact

Questions about the technical specifics of performing the protocol should be directed to and will be fulfilled by the technical contact, Yuriy Shymkiv (ys2605@columbia.edu).

### Materials availability

This study did not generate new unique reagents or materials. All software and datasets used are listed in the key resources table.

### Data and code availability

• The calcium imaging and RNN datasets analyzed in this protocol are publicly available at Dryad: https://doi.org/10.5061/dryad.xsj3tx9q6.
• All original analysis code is publicly available on GitHub: https://github.com/shymkivy/slow_dynamics_protocol.
• Any additional information required to reanalyze the data reported in this paper is available from the lead contact upon request.

## Acknowledgments

We thank Jordan P. Hamm and Sean Escola, who contributed to the work in the associated publication. We also thank past and present members of the Yuste lab for their help and fruitful discussions, and the reviewers for their helpful comments. This work was supported by 10.13039/100000065NINDS (RM1NS132981), 10.13039/100000053NEI (R01EY035248), and 10.13039/100000025NIMH (R01MH115900; F31MH122137, 5T32MH018870).

## Author contributions

Y.S. and R.Y. designed the project. Y.S. conducted experiments, analyzed the data, and wrote the article. Y.S. and R.Y. edited the article. R.Y. assembled and directed the team and provided equipment, resources, and funding.

## Declaration of interests

The authors declare no competing interests.
